# Novel Circovirus in Blood from Intravenous Drug Users, Yunnan, China

**DOI:** 10.3201/eid2905.221617

**Published:** 2023-05

**Authors:** Yanpeng Li, Peng Zhang, Mei Ye, Ren-Rong Tian, Na Li, Le Cao, Yingying Ma, Feng-Liang Liu, Yong-Tang Zheng, Chiyu Zhang

**Affiliations:** Fudan University, Shanghai, China (Y. Li, P. Zhang, L. Cao, Y. Ma, C. Zhang);; Chinese Academy of Sciences, Kunming, China (M. Ye, R.-R. Tian, N. Li, F.-L. Liu, Y.-T. Zheng);; Kunming Medical University, Kunming (N. Li)

**Keywords:** circovirus, IDU, intravenous drug users, viruses, viral metagenomics, emerging virus, transmission, China

## Abstract

We identified a novel circovirus (human-associated circovirus 2 [HuCV2]) from the blood of 2 intravenous drug users in China who were infected with HIV-1, hepatitis C virus, or both. HuCV2 is most closely related to porcine circovirus 3. Our findings underscore the risk for HuCV2 and other emerging viruses among this population.

Viral infections are major threats to human health (*1*,*2*). Because of the advent of high-throughout sequencing, researchers are identifying new viruses with an unprecedented speed (*3*), and continuous attempts at virus discovery and surveillance, especially among regions and populations at high risk for viral outbreaks, play a crucial role in early diagnosis and warning and in the treatment of emerging and reemerging viral infections. 

Circoviruses belong to the *Circoviridae* family, which comprises viruses with circular, single-stranded DNA genomes. The viruses have been identified in a wide range of hosts, including birds, fish, insects, and mammals (*4*). Porcine circovirus (PCV) is the smallest known viral pathogen of *Circoviridae*, and the 4 PCV species that have been recognized (PCV1–PCV4) are widely spread worldwide (*5*). Despite lacking direct evidence of zoonotic transmission of PCVs from animal to human, several studies reported the detection of PCV1 or PCV2 in human samples, and PCV3 was demonstrated as capable of infecting nonhuman primates (*6*). These findings highlight a potential risk for zoonotic transmission of circovirus and the possible presence of novel circoviruses in humans (*7*).

We report the genomic sequences of a novel circovirus from 2 intravenous drug users (IDUs) who were infected with HIV-1, hepatitis C virus, or both. Given the phylogenetic analysis and genetic distance, our finding suggests that this circovirus could represent a new species of human circovirus.

## The Study

IDUs are at high risk for bloodborne viral infections and bear a high burden of the viruses that primarily infect humans (e.g., HIV-1, hepatitis B virus, and HCV). In a previous study, we investigated the plasma viromes of 99 IDUs from several regions of Yunnan, China, which borders with Myanmar (*8*). We found that IDUs have higher plasma viral loads, and we identified (in addition to HIV-1, hepatitis B virus, and HCV) viruses of other vertebrate viral families, including *Anelloviridae*, *Flaviviridae*, *Circoviridae*, *Orthomyxoviridae*, *Papillomaviridae*, *Pneumoviridae*, *Parvoviridae*, and *Kolmioviridae*. Those data highlighted the complex circulation of bloodborne viruses among IDUs. In a subsequent analysis of the blood virome of an IDU patient (patient no. J030) from the city of Dehong in Yunnan Province who was co-infected with HIV-1 and HCV, we detected novel viral sequences related to PCV3 and assembled a complete circular genome (≈2004 nt) (isolate YN09/J030, GenBank accession no. ON226770). This circular viral genome contains 3 main open reading frames (ORFs): 981 nt ORF1 (encoding the potential replication-associated [rep] protein), 645 nt ORF2 (encoding the potential capsid [cap] protein), and 525 nt ORF3 (unknown protein). 

**Figure 1:**
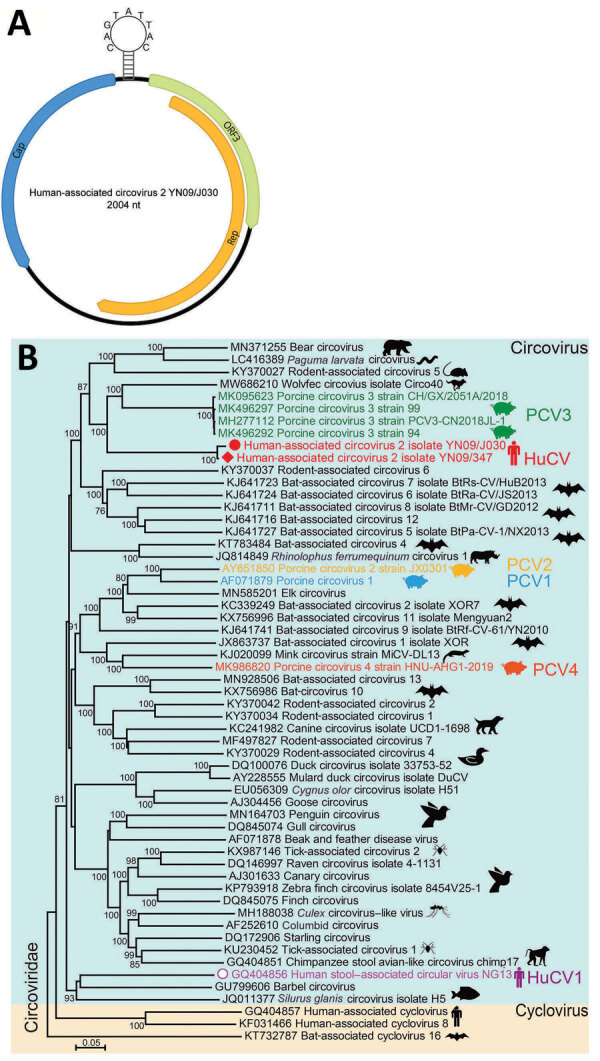
Genomic features of HuCV2 and its phylogenetic relationship with other circoviruses. A) Typical circular genome organization of HuCV2. B) Phylogenetic tree, generated based on the full-length genomic sequences, highlights HuCV2 from 2 patients in China (red circle and red diamond), another circovirus detected in humans (in addition to HuCV1), and the most widely studied known pathogens (PCV1–4). The phylogenetic tree was built in MEGA X (https://www.megasoftware.net) by using the neighbor-joining method. Bootstrap analysis with 1,000 replicates was applied to assess the phylogeny. Scale bar indicates number of nucleotide substitutions per site. Cap, capsid protein; HuCV, human-associated circovirus; ORF3, open reading frame 3; PCV, porcine circovirus; rep, replication-associated protein.

Similar to other circoviruses, a typical stem-loop structure (CAGTATTAC) exists between ORF1 and ORF2 (Figure 1). By using the full-length genomes of isolate YN09/J030 and all the representatives of circovirus, we performed phylogenic analyses to determine the evolutionary relationship of this virus. This novel virus clusters with several PCV3 strains and a circovirus from wolverines (WoCV) to form a phylogenetic clade. The virus shares 60.5% genomic sequence identity with PCV3 and 59.6% with WoCV. The first human-associated circovirus (HuCV1) was previously found in the feces of a child with acute flaccid paralysis in 2010 (*9*). Because of the nonsterile nature of feces, the origin of HuCV1 is unclear, and its association with human remains to be established. We named the newly identified virus human-associated circovirus 2 (HuCV2) (isolate YN09/J030), which we believe represents a new species of human circovirus.

**Figure 2:**
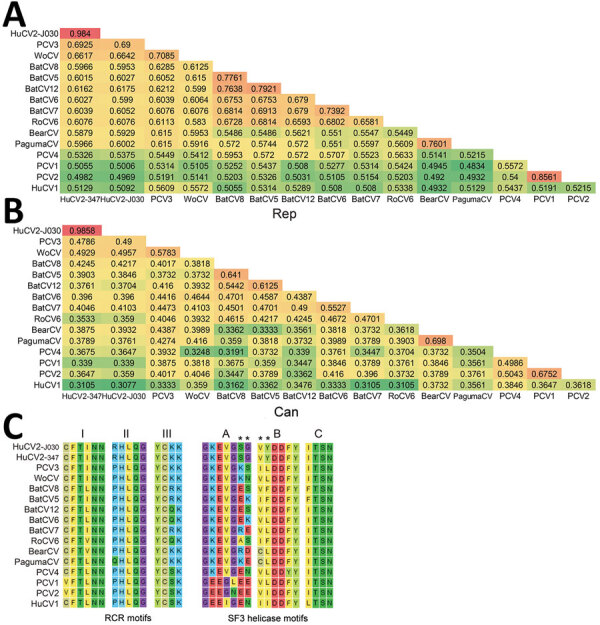
Genetic relationship of HuCV2 from 2 patients in China to other circoviruses. A, B) Pairwise identities of the rep (A) and cap (B) protein of HuCV2 isolate YN09/J030 to other circoviruses. C) Comparison of main residues in the conserved domains of rep protein (RCR and SF3 helicase motifs) between HuCV2 isolates YN09/J030 and YN09/347 and other circoviruses. Asterisks (*) indicate unique residues that are different from PCV3 or WoCV. BatCV, bat-associated circovirus; BearCV, bear-associated circovirus; Cap, capsid protein; HuCV, human-associated circovirus; PagumaCV, paguma circovirus; PCV, porcine circovirus; RCR, rolling circle replication; Rep, replication-associated protein; RoCV, rodent-associated circovirus; SF3, superfamily 3; WoCV, wolverine-associated circovirus.

Both rep and cap regions of HuCV2 clustered with the clade formed by PCV3 and WoCV. HuCV2 rep region shares 69.5% identity at the amino acid level with PCV3 and 66.7% with WoCV; the cap region shares 47% identity at the amino acid level with PCV3 and 50.4% with WoCV (Appendix Figure 1). We identified conserved regions of the rep protein, including the rolling circle replication and superfamily 3 helicase motifs. HuCV2 shares identical rolling circle replication motifs with PCV3 and WoCV, whereas Walker A motifs of HuCV2 superfamily 3 helicase have 2-residue differences with PCV3 and WoCV (Figure 2). Furthermore, we observed a 1-residue difference in HuCV2 Walker B motif from that of PCV3 and WoCV (Figure 2). We detected no recombination signals among HuCV2, PCV3 or WoCV, and circoviruses from outlying lineages, such as bat circoviruses.

To investigate HuCV2 prevalence, we developed a specific quantitative PCR method on the basis of its genomic sequence and screened 568 blood samples collected from IDUs in the same region and in several other cities of Yunnan Province (Appendix Figure 2). We detected HuCV2 sequence (cycle threshold 31) in a second patient (patient no. 347) who was co-infected with HCV. This patient was from Linxiang District of the city of Lincang and had no contact with the first case-patient. We subsequently amplified and sequenced the near full-length genome of the second circovirus isolate (isolate YN09/347, GenBank accession no. OP744467) from this patient’s blood. The second HuCV2 strain shares ≈98.5% sequence identity with isolate YN09/J030 (Figure 2).

Patients J030 had HIV-1 diagnosed in 2005 and patient 347 in 1996 (Table). In our previous study (*10*), we identified both patients as HCV-positive by using quantitative reverse-transcription PCR and next-generation sequencing. Patient J030 did not receive highly active antiretroviral therapy and died in 2010. Patient 347 received the therapy but died of non–AIDS-related causes in 2014.

**Table:** Demographic and clinical characteristics of 2 patients with novel circovirus infection, Yunnan, China*

Characteristic	Patient 1, J030	Patient 2, 347
Sex	Male	Male
Ethnicity	Jingpo	Han
Occupation	Farmer	Farmer
Marriage status	Married	Single
Risk behaviors (drug used)	IDU (heroin)	IDU (heroin)
Syringe or needle sharing	Yes	Yes
Year of sampling	2009	2009
Year of 1^st^ HIV-1 diagnosis	2005	1996
HIV-1†	+	–
HCV†	+	+
HuCV2	+	+
Other infections	–	–
AIDS symptoms	No	No
CD4+ cell count, cells/mm^3^	554	635
HAART	No	Yes
Year of death	2010	2014
Cause of death	Unknown	Non–AIDS-related
HAART	No	Yes

*HAART, highly active antiretroviral therapy; HCV, hepatitis C virus; HuCV2, human-associated circovirus 2; injection drug use; +, positive; –, negative or below detection limit. †Nucleic acid detection (quantitative PCR and next-generation sequencing) of the sample collected in 2009, as described in a previous study (*8*).

## Conclusions

The pathogenicity of PCVs in pigs and their potential threat to several other animals is well known, but the pathogenicity or disease association of other circoviruses is barely known. The presence of this novel circovirus HuCv2 in the blood of 2 IDUs underscore the risk for emerging viruses in this population, although the epidemiology and potential clinical importance of HuCV2 remains to be clarified.

Even though HuCV2 is more closely related to PCV3 than to other circoviruses, the low nucleotide sequence identity (60.5%) and absence of recombination between them indicate that HuCV2 could be a novel circovirus species that circulates in humans. Only 2 IDUs were detected to be HuCV2-positive, implying very low prevalence. However, all the blood samples were collected during 2009–2010, and after >10 years’ storage and repeated freezing and thawing consistent with their experimental purposes, the viral genomes could be degraded. Furthermore, both patients shared needles or syringes with other IDUs (Table) and more likely spread or acquired HIV-1, HCV, and HuCV2 to or from others. Therefore, HuCV2 prevalence might be underestimated. On the other hand, whether the presence of HuCV2 in these 2 patients was associated with their health conditions or their deaths was unclear, and the infectivity and pathogenicity of HuCv2 need to be further determined. Injection drug use and other risk practices are major drivers of new bloodborne viral infections and transmission. The blood samples in our study were collected from the regions of Yunnan Province that border with Myanmar, where extensive co-infection and transmission of bloodborne viruses (e.g., HIV-1 and HCV) among IDUs were reported because of active drug trading, syringe sharing, and high-risk sexual behaviors (*10*,*11*). Thus, even though the 2 cases were reported in 2009, HuCV2 might have spread among IDUs and could be currently circulating in this population of this region, warranting further epidemiologic investigation.

In conclusion, the detection of this novel HuCV2 among IDUs raises question about its origin, prevalence, and pathogenicity in humans. In addition, HuCV2 might circulate in animals, and detection in humans may have resulted from spillover through unknown routes. Thus, screening for HuCV2 infection in animals, especially those that closely interact with humans, could be considered to determine its potential origin and host range.

Appendix Additional information about novel circovirus in blood from intravenous drug users, Yunnan, China.
