# Supplementary material for: Novel Circovirus in Blood from Intravenous Drug Users, Yunnan, China
Source: Emerg Infect Dis. 2023 May;29(5):1015–9. doi: 10.3201/eid2905.221617 (PMC10124637; doi:10.3201/eid2905.221617)
Supplement: Appendix — Additional information about novel circovirus in blood from intravenous drug users, Yunnan, China. [file 22-1617-Techapp-s1.pdf]

Article DOI: https://doi.org/10.3201/eid2905.221617

Novel Circovirus in Blood from Intravenous Drug Users, Yunnan, China

Appendix

Materials and Methods

Sample Processing, Library Construction, And Sequencing

The plasma virome was investigated and reported in our previously study (*1*). Briefly, 200 uL of plasma was thawed on ice and homogenized, and the suspension was clarified by centrifugation at 12,000 g for 15 min. Background materials were reduced by filtration through a 0.45 um sterile filter (Costar Spin-X centrifuge tube filters, Corning, USA) and the treatment with a cocktail of nucleases (15U Turbo DNase, 20U Benzonase, and 20U RNase I, 2 h at 37°C). Total nucleic acids were extracted using QIAamp MinElute virus kit (Qiagen, Germany), and were amplified using a random-amplification approach (REPLI-g Single Cell WTA kit, Qiagen, Germany). The amplified products were purified by QIAquick PCR purification kit (Qiagen, Germany). DNA libraries were prepared using NEBNext UltraII FS DNA library Prep Kit (Illumina, USA), quantified by Qubit3.0 (Invitrogen, USA) and sequenced on the Illumina Novaseq platform (Illumina, USA) with 2 × 150-bp paired reads.

Virome Bioinformatic Analyses

In brief, sequencing adaptors and low-quality sequences were removed using Trimmomatic v.0.38 (*2*). The remaining high-quality reads were de novo assembled using Megahit v.1.1.3 (*3*). Both singlets and assembled contigs were mapped against viral nucleic acid database selected from the NCBI nt database (based on annotation taxonomy in Virus Kingdom) using BLASTn (E<10–10). Reads and contigs with no hits from the previous step were further mapped against viral protein database with BLASTx (E<10–5) (DIAMOND v.0.9.24) (*4*). False positives of the candidate viral hits were removed by mapping all viral hits against the NCBI nt database (ftp://ftp.ncbi.nih.gov/blast/db). Viral reads and contigs were manually checked to exclude potential artifacts.

Genomic Reconstruction and Phylogenetic Analyses

The full-length genome of HuCV2 was assembled and verified using the Geneious R11 program (*5*). To investigate the evolutionary relationship of HuCV2, full-length genome, Rep and Cap regions of this virus and all the representative sequences from *Circoviridae* were used to generate the phylogenetic trees. Viral nucleic acid sequences were aligned using MAFFT (*6*). Alignments were manually checked to avoid miss matches or ambiguous regions. Model test program was used to determine the best substitution model. Neighbor-joining (NJ) and Maximum-likelihood (ML) phylogenetic trees based on nucleotide sequences were generated using the bootstrap method (1 000 times) in MEGA X.

Q-PCR Detection of HuCV2 from Blood Samples

In total, 568 blood samples of IDUs collected from the same region as well as several other cities of Yunnan Province were used for the virus screening. The viral nucleic acids were extracted using the Magnetic Viral DNA/RNA Kit (TIANGEN, BIOTECH, China) according to the manufacturer's instructions. Specific qPCR primers were designed: Forward, 5′-GGTTTTATGCGTGCTCACTTAG-3′; Reverse, 5′-TGCTGTCTCCAAATCCACTC-3′; Probe, 5′-FAM-TCTCCAATCTCCAGGTAATCCCCGT-BHQ1–3′. Program: 40 cycles of 95°C for 15s and 60°C for 30 s.

References

1. Li Y, Cao L, Ye M, Xu R, Chen X, Ma Y, et al. Plasma virome reveals blooms and transmission of anellovirus in intravenous drug users with HIV-1, HCV, and/or HBV infections. Microbiol Spectr. 2022;10:e0144722. PubMed https://doi.org/10.1128/spectrum.01447-22

2. Bolger AM, Lohse M, Usadel B. Trimmomatic: a flexible trimmer for Illumina sequence data. Bioinformatics. 2014;30:2114–20. PubMed https://doi.org/10.1093/bioinformatics/btu170

3. Fu L, Niu B, Zhu Z, Wu S, Li W. CD-HIT: accelerated for clustering the next-generation sequencing data. Bioinformatics. 2012;28:3150–2. PubMed https://doi.org/10.1093/bioinformatics/bts565

4. Buchfink B, Xie C, Huson DH. Fast and sensitive protein alignment using DIAMOND. Nat Methods. 2015;12:59–60. PubMed https://doi.org/10.1038/nmeth.3176

5. Kearse M, Moir R, Wilson A, Stones-Havas S, Cheung M, Sturrock S, et al. Geneious Basic: an integrated and extendable desktop software platform for the organization and analysis of sequence data. Bioinformatics. 2012;28:1647–9. PubMed https://doi.org/10.1093/bioinformatics/bts199

6. Katoh K, Rozewicki J, Yamada KD. MAFFT online service: multiple sequence alignment, interactive sequence choice and visualization. Brief Bioinform. 2019;20:1160–6. PubMed https://doi.org/10.1093/bib/bbx108

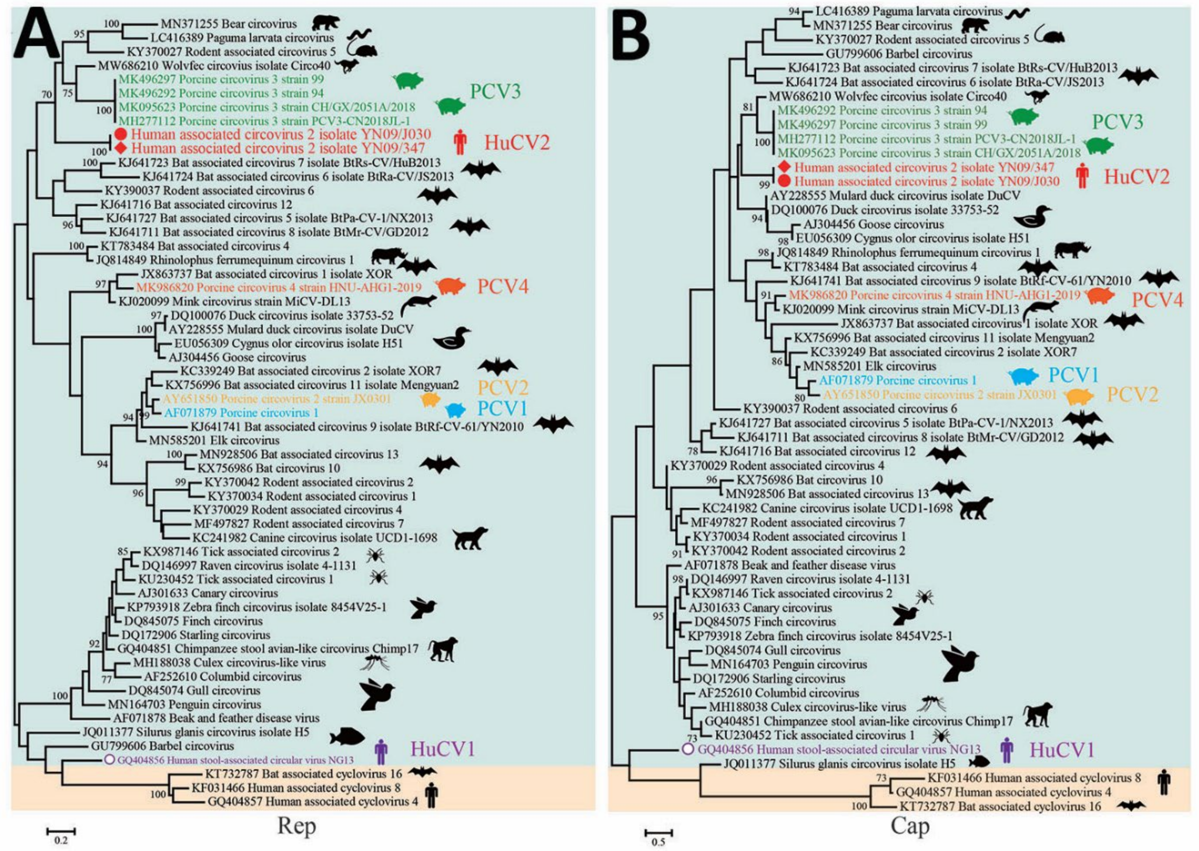

Appendix Figure 1. Phylogenetic trees of rep and cap region of circovirus. The trees were generated based on the full-length sequences of rep (A) and cap (B) regions. Phylogenetic trees were built in MEGA X using the maximum-likelihood (ML) method under GTR+G+I model.

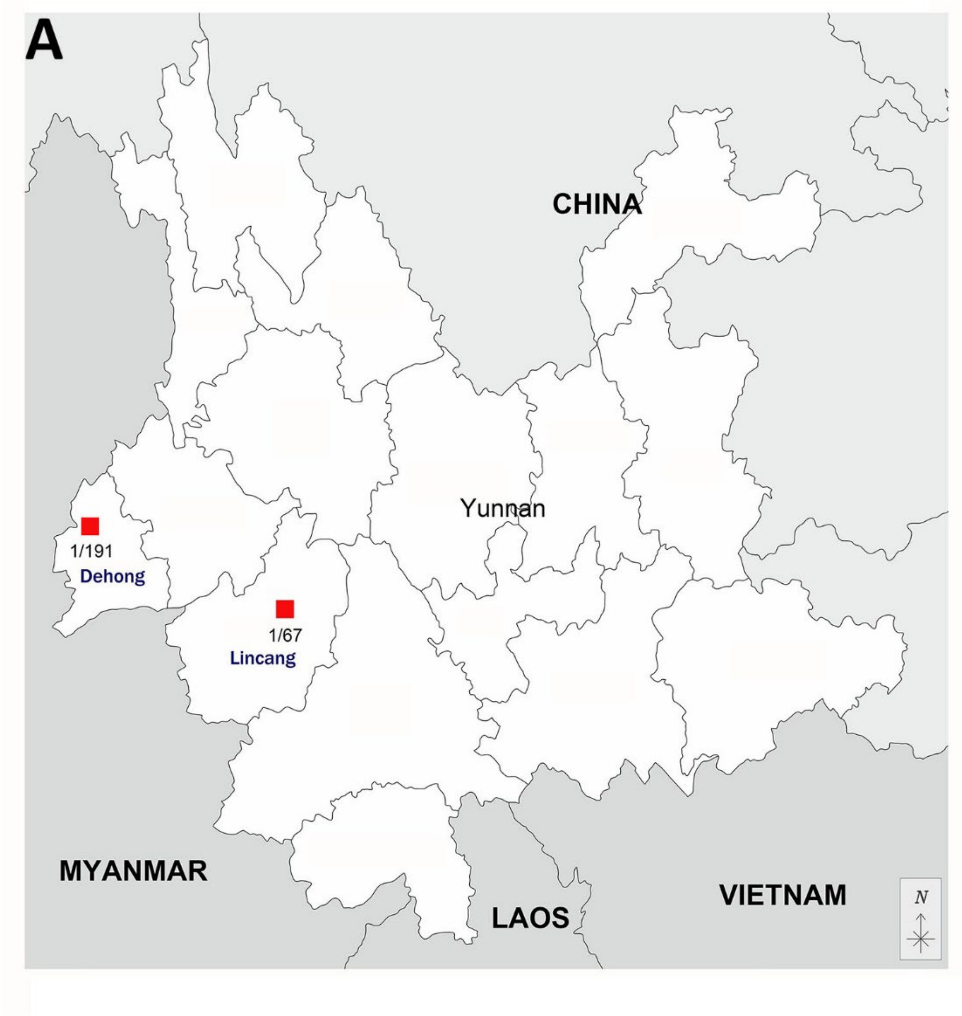

Appendix Figure 2. Geographic Geographical location of the two IDU cases that HuCV2 was found. HuCV2 was found in 1/191 blood samples from Dehong, and 1/67 samples from Linxiang of Yunnan, China.
